# Potentials of Raspberry Ketone as a Natural Antioxidant

**DOI:** 10.3390/antiox10030482

**Published:** 2021-03-18

**Authors:** Sung Ho Lim, Chang-Ik Choi

**Affiliations:** 1 BK21 FOUR Team and Integrated Research Institute for Drug Development, College of Pharmacy, Dongguk University-Seoul, Goyang 10326, Korea; 93sho617@naver.com; 2 Integrated Research Institute for Drug Development, College of Pharmacy, Dongguk University-Seoul, Goyang 10326, Korea

**Keywords:** raspberry ketone, natural antioxidant, total antioxidant capacity, antioxidant enzyme activity, lipid peroxidation

## Abstract

Oxidative stress is closely linked to various diseases, and many studies have been conducted to determine how to reduce this stress. In particular, efforts are being made to find potential antioxidants from natural products. Studies have shown that raspberry ketone (RK; 4-(4-hydroxyphenyl)-2-butanone) has various pharmacological activities. This review summarizes the antioxidant activities of RK and their underlying mechanisms. In several experimental models, it was proven that RK exhibits antioxidant properties through increasing total antioxidant capacity (TAC); upregulating antioxidant enzymes, such as superoxide dismutase (SOD) and catalase (CAT); and improving lipid peroxidation. In conclusion, research about RK’s antioxidant activities is directly or indirectly related to its other various physiological activities. Further studies at the clinical level will be able to verify the value of RK as an effective antioxidant, functional health food, and therapeutic agent.

## 1. Introduction

Oxidative stress is defined as the imbalance between the generation of free radicals and reactive metabolites, so-called “oxidants” or reactive oxygen species (ROS), and the cell’s ability to neutralize them by antioxidant defense [1]. This imbalance can lead to damage to important biomolecules and cells and produce more lethal reactive molecules, potentially affecting the whole organism [2]. ROS initiate oxidation both in vitro and in vivo as the main resources of primary catalysts, causing numerous diseases and disorders [3,4]. Thus, over the past decade, the beneficial effects of antioxidants in relation to organisms’ defense mechanisms against pathologies associated with free radical attacks have been an important topic for many scientists around the world [5].

In general, antioxidants are classified as endogenous and exogenous. Endogenous antioxidants consist of enzymes such as superoxide dismutase (SOD), catalase (CAT), and glutathione peroxidase (GSH-Px) [6]. First, SOD is very important because it is the only antioxidant enzyme defense system that decomposes superoxide anion radicals into hydrogen peroxide (H_2_O_2_) [7]. Three distinct forms exist in mammals: intracellular copper–zinc SOD (CuZnSOD), mitochondrial manganese SOD (MnSOD), and extracellular SOD (ECSOD), all of which are expressed in human lungs [8]. Of these, CuZnSOD and MnSOD are known to act as bulk scavengers of superoxide radicals, and ECSOD is present in relatively high levels, due to specific binding to extracellular matrix components in the lung and is considered essential for lung matrix protection [9]. It is closely related to CAT and GSH-Px and breaks down H_2_O_2_ produced by the action of oxidative enzymes, such as SOD, into water [10]. Catalase degrades H_2_O_2_ by conversion between two forms: catalase–ferricatalase (iron coordinated with water) and Compound I (iron complexed with oxygen atoms) [11]. It also binds nicotinamide adenine dinucleotide phosphate (NADPH) as a reducing equivalent, preventing the oxidative inactivation of the enzyme (formation of Compound II) by H_2_O_2_ when it is reduced to water [12]. However, as catalase-peroxidase, CAT also has oxidase activity, which depends on heme and requires oxygen in addition to the electron donor [13]. GSH-Px, another enzyme responsible for H_2_O_2_ reduction, oxidizes reduced glutathione (GSH) to glutathione disulfide, converts peroxides and hydroxyl radicals into non-toxic forms, and then reduces them to GSH by glutathione reductase [14]. Redox homeostasis of the cell is also protected by its complex endogenous antioxidant enzymes such as heme oxygenase and heme-containing peroxidases [15], and non-enzymatic compounds like glutathione, proteins (ferritin, transferrin, ceruloplasmin, and even albumin) and low-molecular weight scavengers, like uric acid, coenzyme Q10, and lipoic acid [6,16].

If endogenous factors cannot provide complete protection against ROS, exogenous antioxidants are needed in the form of foodstuffs, pharmaceuticals, and dietary supplements containing antioxidant compounds [16]. Examples of exogenous antioxidants are α-tocopherol (vitamin E), ascorbic acid (vitamin C), carotenoids (β-carotene), oil lecithins, selenium, drugs such as N-acetylcysteine, and phenolic antioxidants containing stilbene derivatives (resveratrol, phenolic acids, and flavonoids) [17,18]. Alpha-tocopherol is the most active form of vitamin E [19]. It is strategically located within the cell membrane to protect the cell from lipid peroxidation; it can also interfere with radical cascade by forming low-reactivity vitamins that do not attack lipid substrates via hydroxyl groups [20]. Ascorbic acid, one of the most common water-soluble antioxidants, primarily forms semidehydroascorbic acid that scavenges hydroxyl, alkoxyl, and superoxide radical anion and reactive nitrogen species (RNS), providing intracellular and extracellular aqueous antioxidant capacity [21]. The problem with ascorbic acid is its pro-oxidant activity in the presence of transition metal cations. Ascorbate ion can reduce the trivalent iron to its divalent form, which itself oxidizes dehydroascorbate [22]. As a result, metal ions are reduced, re-oxidized, and again reduced in a redox cycle generating reactive oxygen species [23]. The resulting Fenton-type reaction produces reactive oxygen species, namely the highly oxidizing hydroxyl radical and the hydroxyl anion [24]. Carotenoids are pigments synthesized by plants and microorganisms, and β-carotene is known to react with peroxyl, hydroxyl, and superoxide radicals [25]. Carotenoids exhibit antioxidant effects at low partial pressures but have been reported to exhibit oxidation-promoting effects at high oxygen concentrations [26].

Nonetheless, clinical trials supplemented with antioxidants in healthy people have shown contrasting results. While some intervention studies have shown protective effects, others have not described the evidence of benefits or the increased risk of mortality [27]. In addition, the problem of synthetic antioxidants has caused another controversy. For instance, it is argued that the recommended daily doses of vitamins C and E are not sufficient to offset oxidative stress [28]. It is also stipulated that ingestion of large amounts of antioxidant supplements can lead to pro-oxidant effects, or so-called “antioxidant stress” [29]. However, it is suggested that most humans can maintain the set point of oxidative stress regardless of antioxidant supplements; that is, oxidative stress is no longer reduced [30,31]. Providing cells with exogenous antioxidants can delay the absorption of endogenous antioxidants and keep the whole cellular antioxidant potential unchanged. Thus, antioxidant supplements may improve the organism’s ability to suppress oxidative stress that cannot be modified by the intervention of endogenous antioxidant defenses [16].

**Figure 1:**
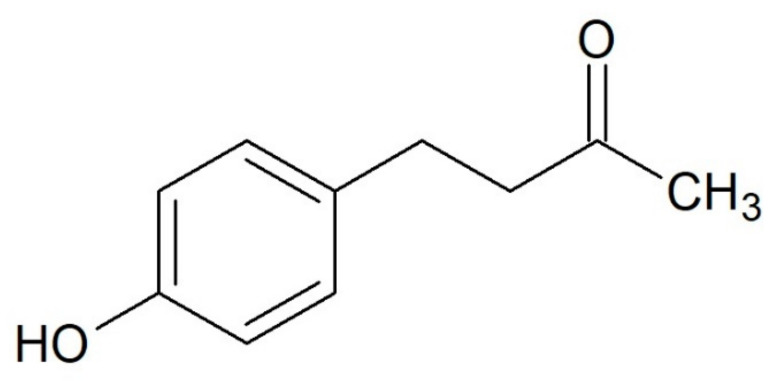
The chemical structure of raspberry ketone (RK).

Raspberry ketone (RK; 4-(4-hydroxyphenyl)-2-butanone) (Figure 1) is an aromatic compound primarily found in red raspberries (*Rubus idaeus* L.) [32]. RK is the key compound responsible for the fruity aroma of raspberries and is widely used as a fragrance and flavoring agent for cosmetics, perfumes, soft drinks, and foods [33]. Although RK content is highest in red raspberries at 0.001–4.20 mg/kg [34,35,36,37,38], it has also been found in small amounts (0.00081–0.7 mg/kg) in a variety of other sources such as yew (*Taxus baccata* L.) [39,40], the lips (labellum) of orchid flowers (*Bulbophyllum apertum* Schltr.) [41], brewed coffee (*Coffea arabica* L.) [42,43], and kiwi berry (*Actinidia arguta* Planch. ex Miq.) [44].

RK was first identified in 1903, and the chemical structure of this compound isolated from raspberries was determined in 1951 [45]. The structure of RK resembles that of capsaicin and synephrine, compounds that have anti-obesity effects and modulate lipid metabolism [46]. When structural similarities of these three compounds have been noted, extensive anti-obesity studies have been conducted with RK [47,48,49]. In addition, RK has numerous pharmacological activities, such as antidiabetic [50], anti-inflammatory [51], antifungal [52,53], gastroprotective [54], skin-whitening [55], hepatoprotective [56,57], anti-androgenic [58], cardioprotective [59], and hair growth promoting [60] properties. Although much research on the antioxidant activity of RK has been reported, reviews are still lacking. Therefore, in this article, we summarize the available information on RK’s antioxidant activities in vitro and in vivo and their mechanisms of action.

## 2. Methodology

The scientific literature (e.g., PubMed, Google Scholar, Web of Science) on the origin, current use/applications, and biological studies of raspberry ketone published in the English language until 2020 were collected. We screened for the different database terms, such as “raspberry ketone”, “biological activity”, “pharmacological activity”, “antioxidant”, “anti-inflammatory”, “anti-obesity”, “antidiabetic”, “anticancer”, and so forth, and in some cases, the combined form of these terms was used for the literature search.

As part of this search, 38 original research articles were found to describe various health-promoting effects of RK so far. Among these, a total of 10 papers have discussed RK’s antioxidant activity and its association with certain chronic diseases. We have comprehensively summarized the study design, result, and its interpretation of each paper.

## 3. RK’s Antioxidant Activities and Their Underlying Mechanisms

Summary of antioxidant activities and their underlying mechanisms are described in Table 1.

### 3.1. Total Antioxidant Capacity (TAC)

The TAC measurement is one of the most frequently used strategies for assessing the free radical–antioxidant balance in biological systems [67]. Clinically relevant antioxidants are divided into two groups: preventive antioxidants, such as CAT, GSH-Px, and metal binding proteins, which prevent reactive radical production by reducing hydroperoxides to molecular species without forming free radicals or sequestering transition metals (iron and copper) and chain-breaking antioxidants, such as SOD, GSH, uric acid, and bilirubin, which can trap free radicals directly and disrupt chain-propagation reactions [68]. Numerous methods have been proposed to quantify and numerically express TAC in natural products and biological fluids, such as blood, cerebrospinal fluid, amniotic fluid, tears, plasma, urine, and seminal fluid [69]. The principles of TAC measurement gleaned from the literature are as follows. In solution, the standardized Fe–EDTA complex reacts with hydrogen peroxide by the Fenton-type reaction to form hydroxyl radicals (•OH). These reactive oxygen species break down benzoate and release thiobarbituric acid reactive substances (TBARS). Antioxidants in human body fluid samples inhibit the production of TBARS; the inhibition of color development, defined as antioxidative activity, can be measured using a spectrophotometer [70,71,72]. Aforementioned methods for TAC measurement are not reproducible and cannot be used in many laboratories, due to the instability of the substrate used for lipid peroxidation and TBARS formation. Nevertheless, researchers have suggested that this method of measuring TAC in serum and other biological fluids is very simple and fast [73].

Mohamed et al. [61] calorimetrically measured TAC activity using model animals with a weight gain of at least 30%, and they were classified into five groups (*n* = 10 for each group); O-AD (obese rats maintained on a high-fat diet during the entire study), OCR (calorie-restricted obese rats), ORK (obese rats treated orally with RK (44 mg/kg/day)), OCRRK (obese rats switched to a calorie-restricted diet and treated with RK), and OCRD (obese rats switched to a calorie-restricted diet and treated orally with orlistat (a commercial over-the-counter drug for obesity management, 10 mg/kg/day)). After the six-week treatments, the TAC value of the O-AD group decreased to 0.423 ± 0.03 mg/dL compared with the normal control group (0.998 ± 0.12 mg/dL), suggesting that oxidative stress was observed in obese rats. After a calorie-restricted diet alone, or with added RK or orlistat for six weeks, the ORK group had significantly increased TAC (0.7 ± 0.05 mg/dL) compared with the O-AD group. In addition, the TAC value of the OCRRK group was 1.12 ± 0.13 mg/dL, which was significantly higher than the OCR group (0.66 ± 0.04 mg/dL). The TAC value of the OCRD group showed no significant change.

Khan et al. [59] measured TAC in the serum samples of Wistar rats. In this study, the toxic control (85 mg/kg isoproterenol; subcutaneously) showed a significant reduction in TAC when compared with the vehicle control (0.1 mL saline solution; subcutaneously). In the rats treated with RK (100 and 200 mg/kg/day; orally) significantly higher TAC were observed compared with the toxic control group. Excessive production of ROS and lipid peroxidation are closely related to myocardial infarction pathology [74]. ROS cause membrane injury and cell death, resulting in a vulnerable myocardial condition [75]. The results that show the ability of RK to improve antioxidant defense system is evidence of its effectiveness in preventing myocardial infarction.

Another study conducted by Fouad et al. [57] evaluated whether RK exerts a protective effect against liver injury in vivo and assessed the underlying mechanisms using the carbon tetrachloride (CCl_4_)-induced hepatotoxicity model. In general, oxidative stress is thought to be a major cause of toxicity in CCl_4_-exposed hepatocytes, mediated by the production of free radical metabolites of CCl_4_. [76]. Compared with the control group, CCl_4_-induced damage caused oxidative stress, which was accompanied by a significant decrease in TAC. The group treated with four different concentrations (25, 50, 100, and 200 mg/kg) of RK reversed the effects of CCl_4_ on TAC in a dose-dependent manner. It was the first study to suggest that RK protects the liver from CCl_4_-induced toxicity and can be considered a potential antioxidant for use against hepatotoxicity.

### 3.2. Antioxidant Enzyme Activity

As mentioned above, SOD is a key antioxidant enzyme that acts as the first line of defense against ROS in the antioxidant pathway [77]. The role of the defense mechanisms mentioned is to effectively neutralize free radicals and ROS to prevent lipid peroxidation, protein modification, and loss of biological function induced by ROS accumulation [78]. SOD converts the superoxide anion radical into less reactive hydrogen peroxide and molecular oxygen [79]. After that, hydrogen peroxide can easily pass through the cell membrane and is converted to H_2_O by CAT, another important antioxidant enzyme located mainly in the peroxisome and the inner mitochondrial membrane [80]. Thus, SOD and CAT can eliminate excess ROS and maintain an antioxidant balance, consequently protecting animals from oxidative stress [77].

In the study conducted by Wang et al. [56], rats were randomly divided into five groups after being fed a normal diet for one week. The normal control (NC) group was fed a normal diet for eight weeks, and the model control (MC) group was fed a high-fat diet. The three groups receiving RK were first fed a high-fat diet for four weeks, and then given intragastric RK at 0.5% (low-dose, RKL), 1% (middle-dose, RKM), or 2% (high-dose, RKH). The level of SOD was determined using an enzyme-linked immunosorbent assay. As a result, SOD activity in the liver homogenates of the MC group was decreased compared with that of the NC group. SOD activity increased in all RK groups compared with the MC group, especially in the RKM and RKH groups, in which they showed significant differences. This result indicated that RK can protect against oxidative stress. 

Khan et al. [62] reported on cardiac protection by RK against isoproterenol (ISO) induced myocardial infarction in rats. In this study, ROS caused by ISO generated severe oxidative stress, which resulted in a decrease in myocardial injury and cardiac damage. The levels of SOD and CAT in myocardial tissue were measured by the pyrogallol-autoxidation inhibition method [81] and catalytic activity [82], respectively. The group receiving 50 mg/kg RK showed increased levels of SOD and CAT compared with the untreated group, in which toxicity had been induced by ISO, although these differences were not significant. In the group treated with 100 and 200 mg/kg RK, SOD and CAT levels were significantly increased in a dose-dependent manner. The authors noted that the improvement of this antioxidant defense system may be attributable to the effect of increased peroxisome proliferator-activated receptor-α (PPAR-α) levels [56]. Increased PPAR-α binds to PPAR-responsive elements (PPREs) in the promoter regions of several antioxidant genes and consequently promotes the expression of antioxidant enzymes, such as CAT and SOD, reducing oxidative stress [83]. This study showed that RK is an effective and promising drug for the treatment of myocardial infarction by its ability to reduce oxidative stress and inflammatory reactions and maintain the lipid profile.

Hamdy et al. [63] conducted a study to evaluate the hepatoprotective effect of raspberry ketones on the oxidative stress induced by acrylamide (AA) in rats. In this experiment, rats were allocated randomly into four groups: Group I (control group); Group II (toxicity induced by AA (5 mg/kg/day)); Group III (treated with RK alone (6 mg/kg/day)); Group IV (treated with both AA and RK). Compared with the SOD and CAT levels of the Group I (150.2 ± 11.4 U/mL and 5.92 ± 0.87 U/mL, respectively), Group II exhibited a reduction in these values by half (75.65 ± 5.87 U/mL and 2.57 ± 0.64 U/mL, respectively). In Group IV, both antioxidant enzyme activities, which were deteriorated by AA, were restored by RK treatment (128.26 ± 6.34 U/mL and 4.13 ± 0.45 U/mL, respectively). Nevertheless, RK alone had no effect on SOD and CAT levels, as shown in Group III (149.5 ± 5.92 U/mL and 5.96 ± 0.96 U/mL, respectively). The authors concluded that RK may have a potent antioxidant capacity that increases both SOD and CAT to counteract free radical production, due to the toxicity induced by AA.

Mohamed et al. [64] aimed to investigate the possible protective effects of RK on lung toxicity induced by cyclophosphamide (CP) in mice. The mice were randomly divided into six groups: Group 1 (control group); Group 2 (received a single intraperitoneal (IP) dose of CP (150 mg/kg)); Groups 3–6 (pre-treated orally with four different doses of RK (25, 50, 100, and 200 mg/kg) for 14 days, before the IP administration of CP). In this study, the SOD and CAT levels in lung tissue were significantly decreased in Group 2 (40.51 ± 3.72 and 59.86 ± 4.72 U/mg protein, respectively) compared with those in Group 1 (70.53 ± 1.87 and 105.87 ± 3.83 U/mg protein, respectively). However, the administration of RK prior to CP administration significantly increased SOD and CAT levels in a dose-dependent manner. Through this research, it was confirmed that RK pre-treatment could remove free radicals and protect lung tissue from CP-induced toxicity by improving changes in oxidative stress biomarkers.

### 3.3. Lipid Peroxidation

Lipid peroxidation is an autocatalytic process commonly resulting from cell death, which causes peroxidative tissue damage involved in the toxicity of inflammation, cancer, and aging. Malondialdehyde (MDA) is an indicator of lipid peroxidation as one of the final products of this process. It is a product of free oxygen radicals formed during oxidative denaturation [84]. GSH is a tripeptide synthesized and found at high concentrations (mM level) in cells [85]. It removes peroxynitrite and hydroxyl radicals and converts hydrogen peroxide into water [86]. Reduced GSH is the active form produced by GSH reductase, which more accurately reflects its antioxidant effect than total GSH [87,88]. In addition, GSH is extensively involved in the cellular clearance of H_2_O_2_ and other hydroperoxides, a reaction catalyzed by various enzymes such as GSH-Px, peroxiredoxin (Prx), glutathione S-transferase (GST) and several glutaredoxin (Grx) isoforms [89]. Of these, GSH-Px is present at the highest levels in the cytoplasm and mitochondria of the liver and catalyzes the reaction of GSH and hydroperoxides to form the reduction products glutathione disulfide and hydroperoxides [90]. Therefore, low GSH-Px activity is an early indicator of impaired pro-oxidant/antioxidant balances, and GSH-Px measurement is especially important in patients experiencing oxidative stress [91].

Mehanna et al. [65] investigated the mechanism by which RK affects the expression of various adipokines to improve hyperlipidemia and insulin resistance in obesity-induced rats with a high-fat diet. In this study, MDA and reduced GSH were determined using a colorimetric method. The obese group had four-fold lower GSH activity and five-fold increased MDA levels compared with the normal group. High-fat diets have been consistently reported to generate oxidative stress, due to the pressure of large body masses [92]. In the groups treated with 250 and 500 mg/kg RK, elevated MDA was decreased, and GSH levels were significantly increased, at both RK doses. The authors concluded that RK plays a protective role against oxidative damage, a key component of the pathogenesis of non-alcoholic fatty liver disease (NAFLD) [93,94]. Other studies have also reported the therapeutic potential of RK in rats with NAFLD [56]. 

Attia et al. [66] studied the effects of RK on redox imbalance in obese insulin resistant rats. The animals used in the experiment were sacrificed using a lethal dose of thiopental (200 mg/kg) after blood collection. Later, both the epididymal (EAT) and visceral (VAT) adipose tissues were excised and divided into two different parts. Oxidative stress was induced in the group fed a high-fat fructose diet for 12 weeks, gene expression of Nrf-2 was significantly downregulated, and GSH was markedly decreased in adipose tissue. Nrf-2 is an important modulator that protects cells from stressors, such as endogenous substances, radiation, environmental toxins, and ROS [95]. In addition, MDA had also increased due to these effects. In the RK (55 mg/kg)-treated group, the GSH content was improved, and lipid peroxidation was reduced, reaching normal levels. The mRNA expression of Nrf-2 in the RK-treated group was significantly increased compared with its expression in the high-fat fructose diet group. 

In addition to TAC, Fouad et al. [57] also measured lipid peroxidation in the CCl_4_-induced hepatotoxicity model. The CCl_4_-treated group demonstrated a larger extent of lipid peroxidation, indicated by increased TBARS in liver tissues compared with the control group. Treatment with RK reversed these results, especially at high concentrations (100 and 200 mg/kg), showing similar TBARS levels to the control group. Moreover, the level of GSH increased in a dose-dependent manner in the RK treatment groups. In previous results, the antioxidant effect of RK varied with dose and was much more potent at higher doses.

To evaluate the possible gastroprotective activity of RK against ethanol-induced gastric ulcers in rats, Badr et al. [54] measured GSH, TBARS, and antioxidant enzymes (GSH-Px and CAT) and evaluated Nrf-2 levels by Western blot analysis. Compared with the normal group (7.1 ± 0.43 µmol/g tissue), the GSH value significantly decreased to 2.8 ± 0.18 µmol/g tissue in the ethanol-treated group. With RK pre-treatment, the GSH value was normalized (7.8 ± 0.21 µmol/g tissue). The TBARS level (32.4 ± 2.7 nmol/g tissue), which was significantly increased by ethanol treatment, decreased by about three times (11 ± 0.5 nmol/g tissue) in the RK treatment group. Moreover, RK significantly improved the levels of GSH-Px and CAT, which were reduced by ethanol treatment. Antioxidant enzymes are protective agents that not only remove free radicals but also prevent damage to the gastric mucosa [96]. Nrf-2 was substantially reduced in the ethanol treated group and this result may have affected oxidative stress and mucosal injury. However, the RK-treated group had significantly increased Nrf-2 expression, indicating the potential gastroprotective activity of RK.

As mentioned above, Hamdy et al. [63] confirmed that RK improves the SOD and CAT levels reduced by AA. In addition, RK treatment significantly decreased the plasma MDA level (Group IV, 13.18 ± 2.618 nmol/mL), which is increased by AA (Group II, 20.83 ± 1.83 nmol/mL). While there was no significant difference in Group III (14.97 ± 0.949 nmol/mL) compared with that in control group (Group I, 13.29 ± 1.12 nmol/mL). This might be associated with the upregulation of the GSH biogenesis, which plays a very important role in protecting cells and tissues from oxidative stress and toxic damage induced by AA.

Likewise, Mohamed et al. [64] also evaluated the MDA level, as a marker for damage to the lipid bilayer of cell membranes, to investigate the effect of CP on oxidative stress. As a result, the MDA level in mice with acute IP administration of CP was significantly increased (group 2, 730.25 ± 16.07 nmol/mg protein) compared with that in control group (group 1, 322.22 ± 9.89 nmol/mg protein). The RK pre-treatment also ameliorated the MDA level in lung tissue in a dose-dependent manner, with the level similar to that in group 1 at the highest dose (200 mg/kg) of RK (group 6, 374.07 ± 5.32 nmol/mg protein).

## 4. Future Perspectives

Oxidative stress is closely related to the occurrence and exacerbation of various chronic diseases; thus, it is particularly important to understand antioxidant activity and its mechanism. It has been reported that RK shows various pharmacological activities including anti-obesity, antidiabetic, antifungal, gastroprotective, hepatoprotective, cardioprotective, and anti-inflammatory activities and can delay the progression of Alzheimer’s disease.

In this review, we confirmed that RK exhibits antioxidant activity through a variety of mechanisms. The various pharmacological activities of RK are associated with antioxidant activity, and this article is the first one that summarizes the extent of RK’s antioxidant activities. However, most of the findings cited in this review are based on in vitro or in vivo studies and do not indicate RK’s effects on humans. Further investigations at the clinical level are needed to evaluate the therapeutic efficacy of RK use in the human body.

More extensive evaluations on the safety and toxicity of RK are also required. According to Flavoring Extract Manufacturers’ Association (FEMA) criteria, RK was given GRAS (Generally Recognized as Safe) status and was approved by the United States Food and Drug Administration (U.S. FDA) for food use (21 CFR 172.515) with no hazard to public health [52]. The reported acute oral lethal dose 50% (LD_50_) in male and female rats for RK was 1.32 g/kg and 1.40 g/kg, respectively. Furthermore, except for a transient reduction of hemoglobin, all rats fed a diet containing 0.1–1.0% RK for 13 weeks showed no significant subacute toxicity including weight gain [52]. On the basis of these data, RK is currently being marketed as a dietary supplement for weight loss, with the recommended doses ranging from 100 to 1400 mg/day [97]. Nevertheless, some researchers still warn of the risk of excessive use of RK supplements because the safety and toxicity of RK has not been fully evaluated, especially in high doses. In a recent study [98], an acute single oral dose of 640 mg/kg RK caused various pathological changes including white adipose tissue atrophy, splenic abnormalities, thymus involution, and elevations of some hematological values in C57BL/6J mice. Further research of RK’s chronic toxicity and its harmful effects on the human body will enable to establish the dosing regimen of RK for safer clinical use.

## Figures and Tables

**Table 1:** Summary of antioxidant activities and underlying mechanisms of raspberry ketone (RK).

Mechanism(s)		Dose	Study Model	Study Result(s)	Ref.
TAC↑		25, 50, 100 and 200 mg/kg	Male Wistar rats induced hepatotoxicity by CCl_4_	Dose-dependent amelioration of TAC by RK treatment, which was decreased by half in the toxic group	[57]
		50, 100 and 200 mg/kg	Wistar albino rats induced cardiotoxicity with ISO	Dose-dependent upregulation of TAC (3.914 ± 0.65 to 9.509 ± 0.84 µmol/L) by RK treatment compared with that in the ISO-treated group (2.598 ± 0.77 µmol/L)	[59]
		44 mg/kg	High-fat diet-fed male Wister albino rats	Increased TAC (0.7 ± 0.05 mg/dL) by RK treatment compared with that in the obese group (0.423 ± 0.03 mg/dL)	[61]
Antioxidant Enzyme Activity	SOD↑	0.5%, 1% or 2%	High-fat diet-fed male female Sprague-Dawley rats	RK high-dose group normalized SOD activity, which was reduced by about 33% with a high-fat diet	[56]
		25, 50, 100 and 200 mg/kg	Male Wistar rats induced hepatotoxicity by CCl_4_	RK at 200 mg/kg normalized the SOD activity, which was dropped by about 50% due to CCl_4_	[57]
		50, 100 and 200 mg/kg	Wistar albino rats induced myocardial by ISO	Significant increases in SOD level in medium- (100 mg/kg) and high-dose (200 mg/kg) of RK groups (58.39 ± 1.28 and 59.52 ± 2.3 U/mg protein, respectively) compared with that in the ISO-treated group (28.77 ± 1.4 U/mg protein)	[62]
		6 mg/kg	Male albino rats induced toxicity by AA	RK co-treatment improved the SOD level (128.26 ± 6.34 U/mL) compared with that in AA-treated group (75.65 ± 5.87 U/mL)	[63]
		25, 50, 100 and 200 mg/kg	Adult male Swiss albino rats induced pulmonary toxicity by CP	Dose-dependent recovery of SOD level (55.32 ± 2.42, 56.28 ± 2.30, 68.36 ± 3.89, and 74.59 ± 2.15 U/mg protein, respectively) by RK pre-treatment against CP-induced toxicity (40.51 U/mg protein)	[64]
	CAT↑	50 mg/kg	Adult male Wistar rats induced gastric lesion by EtOH	CAT was abated with EtOH treatment (3.7 ± 0.07 U/g tissue); RK treatment reversed the CAT level (6.2 ± 0.28 U/g tissue)	[54]
		50, 100 and 200 mg/kg	Wistar albino rats induced myocardial by ISO	Significant increases in CAT level in medium- and high-dose of RK groups (37.44 ± 2.92 and 37.95 ± 2.35 nmol H_2_O_2_/min/mg protein, respectively) compared with that in the ISO-treated group (14.75 ± 1.98 nmol H_2_O_2_/min/mg protein)	[62]
		6 mg/kg	Adult male albino rats induced toxicity by AA	RK co-treatment improved the CAT level (4.13 ± 0.45 U/mL) compared with that in AA-treated group (2.57 ± 0.64 U/mL)	[63]
		25, 50, 100 and 200 mg/kg	Adult male Swiss albino rats induced pulmonary toxicity by CP	Dose-dependent recovery of CAT level (84.31 ± 4.75, 89.31 ± 1.70, 116.32 ± 12.25, and 128.59 ± 9.30 U/mg protein, respectively) by RK pre-treatment against CP-induced toxicity (59.86 ± 4.72 U/mg protein)	[64]
	GSH-Px↑	50 mg/kg	Adult male Wistar rats induced gastric lesion by EtOH	Significant amelioration of GSH-Px activity (262 ± 15.7 U/g tissue) compared with that in the EtOH group (86.8 ± 5.6 U/g tissue)	[54]
Lipid peroxidation	GSH↑	50 mg/kg	Adult male Wistar rats induced gastric lesion by EtOH	Significant decrease in GSH content in the EtOH-treated group (2.8 ± 0.18 µmol/g tissue) compared with that in the control group (7.1 ± 0.43 µmol/g tissue); RK normalized the GSH content (7.8 ± 0.21 µmol/g tissue)	[54]
		25, 50, 100 and 200 mg/kg	Male Wistar rats induced hepatotoxicity by CCl_4_	Dose-dependent recovery in GSH levels by RK treatment, which was reduced by 40% due to CCl_4_	[57]
		50, 100 and 200 mg/kg	Wistar albino rats induced myocardial by ISO	Significant elevations in GSH level in medium- and high-dose of RK groups (3.88 ± 0.08 and 4.07 ± 0.18 µmol GSH/mg protein, respectively) compared with that in the ISO-treated group (0.97 ± 0.15 µmol GSH/mg protein)	[62]
		250 and 500 mg/kg	High-fat diet-fed Male Wistar albino rats	RK treatment 2-fold increased the content of GSH, compared with that in the obese group	[65]
		55 mg/kg	High-fat diet-fed adult male Wistar rats	Normalized GSH content in the RK-treated group, compared with that in the high-fat diet group	[66]
	MDA↓	0.5%, 1% or 2%	High-fat diet-fed male female Sprague-Dawley rats	MDA level in RK high-dose group (36.2798 ± 6.454 nmol/mg of protein) was significantly lower than that in the high-fat diet group (47.9707 ± 3.187 nmol/mg of protein)	[56]
		44 mg/kg	High-fat diet-fed male Wistar albino rats	Decreased MDA level in RK-treated group (29 ± 4.2 m/dL compared with that in the obese group (48.1 ± 5.2 mg/dL)	[61]
		50, 100 and 200 mg/kg	Wistar albino rats induced myocardial by ISO	Significant reductions in MDA level in medium- and high-dose of RK groups (5.84 ± 0.33 and 5.66 ± 0.34 nmol MDA/mg protein, respectively) compared with that in the ISO-treated group (9.40 ± 0.45 nmol MDA/ mg protein)	[62]
		6 mg/kg	Male albino rats induced toxicity by AA	RK co-treatment ameliorated the MDA level (13.18 ± 2.618 nmol/mL) compared with that in AA-treated group (20.83 ± 1.81 nmol/mL)	[63]
		25, 50, 100 and 200 mg/kg	Adult male Swiss albino rats induced pulmonary toxicity by CP	Dose-dependent recovery of MDA level (537.04 ± 13.73, 456.79 ± 5.12, 424.69 ± 6.67 and 374.07 ± 5.32 U/mg protein, respectively) by RK pre-treatment against CP-induced toxicity (730.25 ± 16.07 nmol/mg protein)	[64]
		250 and 500 mg/kg	High-fat diet-fed Male Wistar albino rats	RK treatment reduced MDA by approximately 50% compared with that in the obese group	[65]
		55 mg/kg	High-fat diet-fed adult male Wistar rats	RK treatment group normalized the MDA level	[66]
Nrf-2 ↑		50 mg/kg	Adult male Wistar rats induced gastric lesion by EtOH	Nrf-2 expression in the RK-treated group was increased by 50% compared with that in the EtOH-treated group	[54]
NOXs ↓		50 mg/kg	Adult male Wistar rats induced gastric lesion by EtOH	NOX-1 and NOX-4 expressions in the RK-treated group were abated by about 50% compared with that in the EtOH-treated group	[54]

AA, acrylamide; CAT, catalase; CCl_4_, carbon tetrachloride; CP, cyclophosphamide; EtOH, ethanol; GSH, glutathione; GSH-Px, glutathione peroxidase; H_2_O_2_, hydrogen peroxide; ISO, isoproterenol; MDA, malondialdehyde; NOXs, NADPH oxidases; Nrf-2, nuclear factor erythroid-derived 2-related factor 2; SOD, superoxide dismutase; TAC, total antioxidant capacity. The up arrow (↑) means that the level or expression of each variable has increased, and the down arrow (↓) indicates the decrease in the corresponding value.

## References

[1] Reuter S., Gupta S.C., Chaturvedi M.M., Aggarwal B.B. (2010). Oxidative Stress, Inflammation, and Cancer: How Are They Linked?. Free Radic. Biol. Med..

[2] Persson T., Popescu B.O., Cedazo-Minguez A. (2014). Oxidative Stress in Alzheimer’s Disease: Why Did Antioxidant Therapy Fail?. Oxid. Med. Cell. Longev..

[3] Rackova L., Oblozinsky M., Kostalova D., Kettmann V., Bezakova L. (2007). Free Radical Scavenging Activity and Lipoxygenase Inhibition of Mahonia Aquifolium Extract and Isoquinoline Alkaloids. J. Inflamm..

[4] Chanda S., Dave R. (2009). In Vitro Models for Antioxidant Activity Evaluation and Some Medicinal Plants Possessing Antioxidant Properties: An Overview. Afr. J. Microbiol. Res..

[5] Pisoschi A.M., Negulescu G.P. (2011). Methods for Total Antioxidant Activity Determination: A Review. Biochem. Anal. Biochem..

[6] Rahman K. (2007). Studies on Free Radicals, Antioxidants, and Co-Factors. Clin. Interv. Aging.

[7] Kinnula V.L., Crapo J.D., Raivio K.O. (1995). Generation and Disposal of Reactive Oxygen Metabolites in the Lung. Lab. Investig. J. Tech. Methods Pathol..

[8] Kinnula V.L., Crapo J.D. (2003). Superoxide Dismutases in the Lung and Human Lung Diseases. Am. J. Respir. Crit. Care Med..

[9] Zelko I.N., Mariani T.J., Folz R.J. (2002). Superoxide Dismutase Multigene Family: A Comparison of the CuZn-SOD (SOD1), Mn-SOD (SOD2), and EC-SOD (SOD3) Gene Structures, Evolution, and Expression. Free Radic. Biol. Med..

[10] Kinnula V. (2005). Production and Degradation of Oxygen Metabolites During Inflammatory States in the Human Lung. Curr. Drug Target Inflamm. Allergy.

[11] Birben E., Sahiner U.M., Sackesen C., Erzurum S., Kalayci O. (2012). Oxidative Stress and Antioxidant Defense. World Allergy Organ. J..

[12] Kirkman H.N., Rolfo M., Ferraris A.M., Gaetani G.F. (1999). Mechanisms of Protection of Catalase by Nadph Kinetics and Stoichiometry. J. Biol. Chem..

[13] Vetrano A.M., Heck D.E., Mariano T.M., Mishin V., Laskin D.L., Laskin J.D. (2005). Characterization of the Oxidase Activity in Mammalian Catalase. J. Biol. Chem..

[14] Liguori I., Russo G., Curcio F., Bulli G., Aran L., Della-Morte D., Gargiulo G., Testa G., Cacciatore F., Bonaduce D. (2018). Oxidative Stress, Aging, and Diseases. Clin. Interv. Aging.

[15] Szabo I.L., Kenyeres A., Szegedi A., Szollosi A.G. (2018). Heme Oxygenase and the Skin in Health and Disease. Curr. Pharm. Des..

[16] Poljsak B., Šuput D., Milisav I. (2013). Achieving the Balance between ROS and Antioxidants: When to Use the Synthetic Antioxidants. Oxid. Med. Cell. Longev..

[17] Simona C.L., Sandra A.V., Mirela D., Andreia T., Gabriel Lucian R. (2011). Biosensors Applications on Assessment of Reactive Oxygen Species and Antioxidants. J. Environ. Biosens..

[18] Lindsay D.G., Astley S.B. (2002). European Research on the Functional Effects of Dietary Antioxidants—EUROFEDA. Mol. Aspects Med..

[19] White E., Shannon J.S., Patterson R.E. (1997). Relationship between Vitamin and Calcium Supplement Use and Colon Cancer. Cancer Epidemiol. Biomark..

[20] Descamps-Latscha B., Drüeke T., Witko-Sarsat V. (2001). Dialysis-Induced Oxidative Stress: Biological Aspects, Clinical Consequences, and Therapy. Semin. Dial..

[21] Sung C.-C., Hsu Y.-C., Chen C.-C., Lin Y.-F., Wu C.-C. (2013). Oxidative Stress and Nucleic Acid Oxidation in Patients with Chronic Kidney Disease. Oxid. Med. Cell. Longev..

[22] Du J., Cullen J.J., Buettner G.R. (2012). Ascorbic Acid: Chemistry, Biology and the Treatment of Cancer. Biochim. Biophys. Acta.

[23] Valko M., Morris H., Cronin M.T.D. (2005). Metals, Toxicity and Oxidative Stress. Curr. Med. Chem..

[24] Carr A., Frei B. (1999). Does Vitamin C Act as a Pro-Oxidant under Physiological Conditions?. FASEB J..

[25] El-Agamey A., Lowe G.M., McGarvey D.J., Mortensen A., Phillip D.M., Truscott T.G., Young A.J. (2004). Carotenoid Radical Chemistry and Antioxidant/pro-Oxidant Properties. Arch. Biochem. Biophys..

[26] Rice-Evans C.A., Sampson J., Bramley P.M., Holloway D.E. (1997). Why Do We Expect Carotenoids to Be Antioxidants in Vivo?. Free Radic. Res..

[27] Serafini M. (2006). The Role of Antioxidants in Disease Prevention. Medicine.

[28] Pisoschi A.M., Pop A. (2015). The Role of Antioxidants in the Chemistry of Oxidative Stress: A Review. Eur. J. Med. Chem..

[29] Poljsak B., Milisav I. (2012). The Neglected Significance of “Antioxidative Stress”. Oxid. Med. Cell. Longev..

[30] Cutler R.G., Mattson M.P. (2002). Measuring Oxidative Stress and Interpreting Its Clinical Relevance for Humans. Critical Reviews of Oxidative Stress and Aging.

[31] Cutler R.G. (2002). Genetic Stability, Dysdifferentiation, and Longevity Determinant Genes. Critical Reviews of Oxidative Stress and Aging.

[32] Gallois A. (1982). Quantitative evaluation of raspberry ketone using thin-layer chromatography. Sci. Aliments.

[33] Ulbricht C., Catapang M., Conquer J., Costa D., Culwell S., D’Auria D., Isaac R., Le C., Marini E., Miller A. (2013). Raspberry Ketone: An Evidence-Based Systematic Review by the Natural Standard Research Collaboration. Altern. Complement. Ther..

[34] Honkanen E., Pyysalo T., Hirvi T. (1980). The Aroma of Finnish Wild Raspberries, *Rubus Idaeus*, L.. Z. Lebensm. Unters. Forsch..

[35] Larsen M., Poll L. (1990). Odour Thresholds of Some Important Aroma Compounds in Raspberries. Z. Lebensm. Unters. Forsch..

[36] Larsen M., Poll L., Callesen O., Lewis M. (1991). Relations between the Content of Aroma Compounds and the Sensory Evaluation of 10 Raspberry Varieties (*Rubus Idaeus* L.). Acta Agric. Scand..

[37] Borejsza-Wysocki W., Goers S.K., McArdle R.N., Hrazdina G. (1992). (P-Hydroxyphenyl)Butan-2-One Levels in Raspberries Determined by Chromatographic and Organoleptic Methods. J. Agric. Food Chem..

[38] Paterson A., Kassim A., McCallum S., Woodhead M., Smith K., Zait D., Graham J. (2013). Environmental and Seasonal Influences on Red Raspberry Flavour Volatiles and Identification of Quantitative Trait Loci (QTL) and Candidate Genes. Theor. Appl. Genet..

[39] Fronza G., Fuganti C., Guillou C., Reniero F., Joulain D. (1998). Natural Abundance 2H Nuclear Magnetic Resonance Study of the Origin of Raspberry Ketone. J. Agric. Food Chem..

[40] Fronza G., Fuganti C., Pedrocchi-Fantoni G., Serra S., Zucchi G., Fauhl C., Guillou C., Reniero F. (1999). Stable Isotope Characterization of Raspberry Ketone Extracted from Taxus Baccata and Obtained by Oxidation of the Accompanying Alcohol (Betuligenol). J. Agric. Food Chem..

[41] Tan K.-H., Nishida R. (2005). Synomone Or Kairomone? *Bulbophyllum apertum* Flower Releases Raspberry Ketone To Attract Bactrocera Fruit Flies. J. Chem. Ecol..

[42] Akiyama M., Murakami K., Ikeda M., Iwatsuki K., Wada A., Tokuno K., Onishi M., Iwabuchi H. (2007). Analysis of the Headspace Volatiles of Freshly Brewed Arabica Coffee Using Solid-Phase Microextraction. J. Food Sci..

[43] Akiyama M., Murakami K., Hirano Y., Ikeda M., Iwatsuki K., Wada A., Tokuno K., Onishi M., Iwabuchi H. (2008). Characterization of Headspace Aroma Compounds of Freshly Brewed Arabica Coffees and Studies on a Characteristic Aroma Compound of Ethiopian Coffee. J. Food Sci..

[44] Garcia C.V., Quek S.-Y., Stevenson R.J., Winz R.A. (2011). Characterization of the Bound Volatile Extract from Baby Kiwi (*Actinidia arguta*). J. Agric. Food Chem..

[45] Hugueny P., Dumont B., Ropert F., Belin J.M. (1995). The Raspberry Ketone, a Biotechnological Way for Production. Bioflavor.

[46] Morimoto C., Satoh Y., Hara M., Inoue S., Tsujita T., Okuda H. (2005). Anti-Obese Action of Raspberry Ketone. Life Sci..

[47] Park K.S. (2010). Raspberry Ketone Increases Both Lipolysis and Fatty Acid Oxidation in 3T3-L1 Adipocytes. Planta Med..

[48] Park K.S. (2015). Raspberry Ketone, a Naturally Occurring Phenolic Compound, Inhibits Adipogenic and Lipogenic Gene Expression in 3T3-L1 Adipocytes. Pharm. Biol..

[49] Leu S.-Y., Chen Y.-C., Tsai Y.-C., Hung Y.-W., Hsu C.-H., Lee Y.-M., Cheng P.-Y. (2017). Raspberry Ketone Reduced Lipid Accumulation in 3T3-L1 Cells and Ovariectomy-Induced Obesity in Wistar Rats by Regulating Autophagy Mechanisms. J. Agric. Food Chem..

[50] Xiong S.-L., Yue L.-M., Lim G.T., Yang J.-M., Lee J., Park Y.-D. (2018). Inhibitory Effect of Raspberry Ketone on α-Glucosidase: Docking Simulation Integrating Inhibition Kinetics. Int. J. Biol. Macromol..

[51] Jeong J.B., Jeong H.J. (2010). Rheosmin, a Naturally Occurring Phenolic Compound Inhibits LPS-Induced INOS and COX-2 Expression in RAW264.7 Cells by Blocking NF-ΚB Activation Pathway. Food Chem. Toxicol..

[52] Opdyke D.L.J. (1978). 4-(p-Hydroxyphenyl)-2-Butanone. Food Cosmet. Toxicol..

[53] Vaughn S.F., Spencer G.F., Shasha B.S. (1993). Volatile Compounds from Raspberry and Strawberry Fruit Inhibit Postharvest Decay Fungi. J. Food Sci..

[54] Badr A.M., EL-Orabi N.F., Ali R.A. (2019). The Implication of the Crosstalk of Nrf2 with NOXs, and HMGB1 in Ethanol-Induced Gastric Ulcer: Potential Protective Effect Is Afforded by Raspberry Ketone. PLoS ONE.

[55] Lin C.-H.V., Ding H.-Y., Kuo S.-Y., Chin L.-W., Wu J.-Y., Chang T.-S. (2011). Evaluation of in Vitro and in Vivo Depigmenting Activity of Raspberry Ketone from Rheum Officinale. Int. J. Mol. Sci..

[56] Wang L., Meng X., Zhang F. (2012). Raspberry Ketone Protects Rats Fed High-Fat Diets Against Nonalcoholic Steatohepatitis. J. Med. Food.

[57] Fouad D., Badr A., Attia H.A. (2019). Hepatoprotective Activity of Raspberry Ketone Is Mediated via Inhibition of the NF-ΚB/TNF-α/Caspase Axis and Mitochondrial Apoptosis in Chemically Induced Acute Liver Injury. Toxicol. Res..

[58] Ogawa Y., Akamatsu M., Hotta Y., Hosoda A., Tamura H. (2010). Effect of Essential Oils, Such as Raspberry Ketone and Its Derivatives, on Antiandrogenic Activity Based on in Vitro Reporter Gene Assay. Bioorg. Med. Chem. Lett..

[59] Khan V., Sharma S., Bhandari U., Sharma N., Rishi V., Haque S.E. (2019). Suppression of Isoproterenol-Induced Cardiotoxicity in Rats by Raspberry Ketone via Activation of Peroxisome Proliferator Activated Receptor-α. Eur. J. Pharmacol..

[60] Harada N., Okajima K., Narimatsu N., Kurihara H., Nakagata N. (2008). Effect of Topical Application of Raspberry Ketone on Dermal Production of Insulin-like Growth Factor-I in Mice and on Hair Growth and Skin Elasticity in Humans. Growth Horm. IGF Res..

[61] Mohamed H.E., Abo-ELmatty D.M., Mesbah N.M., Saleh S.M., Ali A.-M.A., Sakr A.T. (2018). Raspberry Ketone Preserved Cholinergic Activity and Antioxidant Defense in Obesity Induced Alzheimer Disease in Rats. Biomed. Pharmacother..

[62] Khan V., Sharma S., Bhandari U., Ali S.M., Haque S.E. (2018). Raspberry Ketone Protects against Isoproterenol-Induced Myocardial Infarction in Rats. Life Sci..

[63] Hamdy S.M., El-Khayat Z., Farrag A.R., Sayed O.N., El-Sayed M.M., Massoud D. (2020). Hepatoprotective Effect of Raspberry Ketone and White Tea against Acrylamide-Induced Toxicity in Rats. Drug Chem. Toxicol..

[64] Mohamed M.T., Zaitone S.A., Ahmed A., Mehanna E.T., El-Sayed N.M. (2020). Raspberry Ketones Attenuate Cyclophosphamide-Induced Pulmonary Toxicity in Mice through Inhibition of Oxidative Stress and NF-ΚB Pathway. Antioxidants.

[65] Mehanna E.T., Barakat B.M., ElSayed M.H., Tawfik M.K. (2018). An Optimized Dose of Raspberry Ketones Controls Hyperlipidemia and Insulin Resistance in Male Obese Rats: Effect on Adipose Tissue Expression of Adipocytokines and Aquaporin. Eur. J. Pharmacol..

[66] Attia R.T., Abdel-Mottaleb Y., Abdallah D.M., El-Abhar H.S., El-Maraghy N.N. (2019). Raspberry Ketone and Garcinia Cambogia Rebalanced Disrupted Insulin Resistance and Leptin Signaling in Rats Fed High Fat Fructose Diet. Biomed. Pharmacother..

[67] Fraga C.G., Oteiza P.I., Galleano M. (2014). In Vitro Measurements and Interpretation of Total Antioxidant Capacity. Biochim. Biophys. Acta BBA Gen. Subj..

[68] Woodford F.P., Whitehead T.P. (1998). Is Measuring Serum Antioxidant Capacity Clinically Useful?. Ann. Clin. Biochem..

[69] Bartosz G. (2010). Non-Enzymatic Antioxidant Capacity Assays: Limitations of Use in Biomedicine. Free Radic. Res..

[70] Gutteridge J.M.C., Maidt L., Poyer L. (1990). Superoxide Dismutase and Fenton Chemistry. Reaction of Ferric-EDTA Complex and Ferric-Bipyridyl Complex with Hydrogen Peroxide without the Apparent Formation of Iron(II). Biochem. J..

[71] Winterbourn C.C. (1979). Comparison of Superoxide with Other Reducing Agents in the Biological Production of Hydroxyl Radicals. Biochem. J..

[72] Yamazaki I., Piette L.H. (1990). ESR Spin-Trapping Studies on the Reaction of Fe^2+^ Ions with H_2_O_2_-Reactive Species in Oxygen Toxicity in Biology. J. Biol. Chem..

[73] Koracevic D. (2001). Method for the Measurement of Antioxidant Activity in Human Fluids. J. Clin. Pathol..

[74] Li H., Xie Y.-H., Yang Q., Wang S.-W., Zhang B.-L., Wang J.-B., Cao W., Bi L.-L., Sun J.-Y., Miao S. (2012). Cardioprotective Effect of Paeonol and Danshensu Combination on Isoproterenol-Induced Myocardial Injury in Rats. PLoS ONE.

[75] Hu A., Jiao X., Gao E., Koch W.J., Sharifi-Azad S., Grunwald Z., Ma X.L., Sun J.-Z. (2006). Chronic β-Adrenergic Receptor Stimulation Induces Cardiac Apoptosis and Aggravates Myocardial Ischemia/Reperfusion Injury by Provoking Inducible Nitric-Oxide Synthase-Mediated Nitrative Stress. J. Pharmacol. Exp. Ther..

[76] Zhang J., Shi L., Xu X., Huang S., Lu B., Ji L., Wang Z. (2014). Therapeutic Detoxification of Quercetin against Carbon Tetrachloride-Induced Acute Liver Injury in Mice and Its Mechanism. J. Zhejiang Univ. Sci. B.

[77] Yu H., Deng W., Zhang D., Gao Y., Yang Z., Shi X., Sun J., Zhou J., Ji H. (2017). Antioxidant Defenses of Onychostoma Macrolepis in Response to Thermal Stress: Insight from MRNA Expression and Activity of Superoxide Dismutase and Catalase. Fish Shellfish Immunol..

[78] Lewandowski Ł., Kepinska M., Milnerowicz H. (2018). Inhibition of Copper-Zinc Superoxide Dismutase Activity by Selected Environmental Xenobiotics. Environ. Toxicol. Pharmacol..

[79] Fridovich I. (1986). Biological Effects of the Superoxide Radical. Arch. Biochem. Biophys..

[80] Mruk D.D., Silvestrini B., Mo M., Cheng C.Y. (2002). Antioxidant Superoxide Dismutase—A Review: Its Function, Regulation in the Testis, and Role in Male Fertility. Contraception.

[81] Marklund S., Marklund G. (1974). Involvement of the Superoxide Anion Radical in the Autoxidation of Pyrogallol and a Convenient Assay for Superoxide Dismutase. Eur. J. Biochem..

[82] Claiborne A.L. (1986). Catalase Activity. CRC Handbook of Methods for Oxygen Radical Research.

[83] Schrader M., Fahimi H.D. (2006). Peroxisomes and Oxidative Stress. Biochim. Biophys. Acta BBA Mol. Cell Res..

[84] Ohkawa H., Ohishi N., Yagi K. (1979). Assay for Lipid Peroxides in Animal Tissues by Thiobarbituric Acid Reaction. Anal. Biochem..

[85] Meister A., Anderson M.E. (1983). Glutathione. Annu. Rev. Biochem..

[86] Aldini G., Altomare A., Baron G., Vistoli G., Carini M., Borsani L., Sergio F. (2018). N-Acetylcysteine as an Antioxidant and Disulphide Breaking Agent: The Reasons Why. Free Radic. Res..

[87] Mehta A., Singh S., Ganguly N.K. (1998). Impairment of Intestinal Mucosal Antioxidant Defense System during Salmonella Typhimurium Infection. Dig. Dis. Sci..

[88] Chang Y.W., Jang J.Y., Kim Y.H., Kim J.-W., Shim J.-J. (2015). The Effects of Broccoli Sprout Extract Containing Sulforaphane on Lipid Peroxidation and Helicobacter Pylori Infection in the Gastric Mucosa. Gut Liver.

[89] Deponte M. (2013). Glutathione Catalysis and the Reaction Mechanisms of Glutathione-Dependent Enzymes. Biochim. Biophys. Acta.

[90] Paglia D.E., Valentine W.N. (1967). Studies on the Quantitative and Qualitative Characterization of Erythrocyte Glutathione Peroxidase. J. Lab. Clin. Med..

[91] Yang G.Q., Chen J.S., Wen Z.M., Ge K.Y., Zhu L.Z., Chen X.C., Chen X.S. (1984). The Role of Selenium in Keshan Disease. Adv. Nutr. Res..

[92] Zaitone S.A., Barakat B.M., Bilasy S.E., Fawzy M.S., Abdelaziz E.Z., Farag N.E. (2015). Protective Effect of Boswellic Acids versus Pioglitazone in a Rat Model of Diet-Induced Non-Alcoholic Fatty Liver Disease: Influence on Insulin Resistance and Energy Expenditure. Naunyn. Schmiedebergs Arch. Pharmacol..

[93] Noeman S.A., Hamooda H.E., Baalash A.A. (2011). Biochemical Study of Oxidative Stress Markers in the Liver, Kidney and Heart of High Fat Diet Induced Obesity in Rats. Diabetol. Metab. Syndr..

[94] Hsiao P.-J., Hsieh T.-J., Kuo K.-K., Hung W.-W., Tsai K.-B., Yang C.-H., Yu M.-L., Shin S.-J. (2008). Pioglitazone Retrieves Hepatic Antioxidant DNA Repair in a Mice Model of High Fat Diet. BMC Mol. Biol..

[95] Itoh K., Chiba T., Takahashi S., Ishii T., Igarashi K., Katoh Y., Oyake T., Hayashi N., Satoh K., Hatayama I. (1997). An Nrf2/Small Maf Heterodimer Mediates the Induction of Phase II Detoxifying Enzyme Genes through Antioxidant Response Elements. Biochem. Biophys. Res. Commun..

[96] Guzmán-Gómez O., García-Rodríguez R.V., Quevedo-Corona L., Pérez-Pastén-Borja R., Rivero-Ramírez N.L., Ríos-Castro E., Pérez-Gutiérrez S., Pérez-Ramos J., Chamorro-Cevallos G.A. (2018). Amelioration of Ethanol-Induced Gastric Ulcers in Rats Pretreated with Phycobiliproteins of *Arthrospira* (Spirulina) Maxima. Nutrients.

[97] Bredsdorff L., Wedebye E.B., Nikolov N.G., Hallas-Møller T., Pilegaard K. (2015). Raspberry ketone in food supplements—High intake, few toxicity data—A cause for safety concern?. Regul. Toxicol. Pharmacol..

[98] Hao L., Kshatriya D., Li X., Badrinath A., Szmacinski Z., Goedken M.J., Polunas M., Bello N.T. (2020). Acute feeding suppression and toxicity of raspberry ketone [4-(4-hydroxyphenyl)-2-butanone] in mice. Food Chem. Toxiccol..

